# Using Person Fit Statistics to Detect Outliers in Survey Research

**DOI:** 10.3389/fpsyg.2017.00863

**Published:** 2017-05-26

**Authors:** John M. Felt, Ruben Castaneda, Jitske Tiemensma, Sarah Depaoli

**Affiliations:** Psychological Sciences, University of California, MercedMerced, CA, United States

**Keywords:** item response theory, person fit, quality of life, CushingQoL, Cushing's syndrome

## Abstract

**Context:** When working with health-related questionnaires, outlier detection is important. However, traditional methods of outlier detection (e.g., boxplots) can miss participants with “atypical” responses to the questions that otherwise have similar total (subscale) scores. In addition to detecting outliers, it can be of clinical importance to determine the reason for the outlier status or “atypical” response.

**Objective:** The aim of the current study was to illustrate how to derive person fit statistics for outlier detection through a statistical method examining person fit with a health-based questionnaire.

**Design and Participants:** Patients treated for Cushing's syndrome (*n* = 394) were recruited from the Cushing's Support and Research Foundation's (CSRF) listserv and Facebook page.

**Main Outcome Measure:** Patients were directed to an online survey containing the CushingQoL (English version). A two-dimensional graded response model was estimated, and person fit statistics were generated using the *Zh* statistic.

**Results:** Conventional outlier detections methods revealed no outliers reflecting extreme scores on the subscales of the CushingQoL. However, person fit statistics identified 18 patients with “atypical” response patterns, which would have been otherwise missed (*Zh* > |±2.00|).

**Conclusion:** While the conventional methods of outlier detection indicated no outliers, person fit statistics identified several patients with “atypical” response patterns who otherwise appeared average. Person fit statistics allow researchers to delve further into the underlying problems experienced by these “atypical” patients treated for Cushing's syndrome. Annotated code is provided to aid other researchers in using this method.

## Introduction

Detection of outliers on questionnaires is important in any field of research (e.g., psychology, behavioral medicine). Typically, outlier detection techniques, such as box-plots, determine outliers from total scores or subscale scores. While these methods for detecting outliers are the most common approach implemented for identifying “atypical” scores, there are certain forms of outliers that cannot be detected using this method. If research participants have similar total scores to one another, then the traditional approach to outlier detection would deem all participants as being “typical.” However, it may be that a minority of these participants has very different patterns of responses across the individual items. It is important to properly identify these participants, as their patterns may represent substantively interesting differences. In this case, the total scores are not informative since they are comparable across participants. Instead, it is the exact pattern of responses across items that will provide researchers with much richer and precise results for each individual.

Traditional methods of outlier detection will not identify participants with substantively or clinically different item response patterns as being “atypical” because these detection methods are based entirely on total scores. Person-level outliers, which identify “atypical” patterns of responses across items, can be detected from person fit statistics generated from a variety of different approaches including item residual, least squares, and item response theory (IRT) methods (Meijer and Sijtsma, 2001).

In a recent study, Ferrando et al. (2016) demonstrated several approaches for evaluating person-level outliers from a factor analytic approach (i.e., item residual and least squares). Under simulated conditions, their approaches demonstrated good detection rates across multiple conditions. Additionally, they illustrated their approach with an applied example using the Spanish version of Ray's Balanced Dogmatism Scale (BDS). Out of a sample of 346, the authors identified 55 potentially inconsistent responders. Among the aberrant responses, several causes for the inconsistent response were identified including sabotaging, random guessing, and extreme responding. Given that person-level outlier detection is not common practice, Ferrando et al. (2016) provided an effective approach in a free program, FACTOR (Lorenzo-Seva and Ferrando, 2013).

While Ferrando et al. (2016) propose a factor analytic approach for detecting person-level outliers, we discuss an alternative statistical method utilizing IRT. The IRT approaches for detecting person-level outliers rely on likelihood-based statistics to determine the most typical response patterns given a specific model. While there are multitudes of approaches to detecting misfit, the IRT likelihood-based approach to generate person fit statistics is one of the more accessible methods. Person fit statistics can be interpreted similarly to *z*-scores, where extreme scores (e.g., ±3.0) are unlikely to be observed and evidence of person-level outliers. While person fit statistics generated from IRT likelihood-based methods can be an important form of outlier detection, they are rarely used in the applied psychological literature.

Person fit statistics have been described in the methodological literature for decades (e.g., Levine and Rubin, 1979). However, their appearance in the applied literature varies by field. For instance, person fit evaluation has been implemented in the fields of assessment (e.g., Meijer et al., 2016), education (e.g., Pan and Yin, 2017), and personality (e.g., LaHuis et al., 2017). Although there has been some application of person fit statistics within the broad field of Psychology (see e.g., Hays et al., 2000; Emons et al., 2007; Engelhard, 2008; Meijer et al., 2008; Credé, 2010), many sub-fields of Psychology (e.g., health psychology and social psychology) have yet to fully embrace this method. Psychology in particular stands to benefit from these statistical methods, especially since much of the research conducted within the field is survey-based. For example, research looking at depression screening (see e.g., Christensen et al., 2017) or cognition (see e.g., Snyder et al., 2015) could benefit from a more sophisticated approach for identifying outliers compared to the traditional box-plot approach (or akin).

To provide a more in-depth view of one of these sub-fields in Psychology, we conducted a literature review within Health Psychology to assess the use of person fit statistics. Specifically, we targeted some of the premier Health Psychology journals (i.e., *Health Psychology, Psychology and Health, British Journal of Health Psychology, and Psychology, Health, and Medicine*). We identified 102 papers published since 2005, which focused on scale development. Out of these 102 papers, only five implemented IRT-based methods and none mentioned person fit evaluation. This literature review highlights the lack of person fit statistics within a major sub-field of Psychology.

The IRT likelihood-based approach to identify person-level outliers, also known as person fit, can be used in all types of survey research. In the present study, we selected patients with Cushing's syndrome (CS) because there are important clinical implications linked to identifying and interpreting outliers. CS is a rare disorder characterized by chronic overexposure to cortisol (Bista and Beck, 2014). CS is typically caused by an adrenocorticotropic hormone (ACTH) releasing pituitary adenoma, but can also result from ectopic or adrenal tumors, or from chronic exposure to high doses of glucocorticoid steroids (Lacroix et al., 2015). Patients experience a number of physical and psychological symptoms that typically improve upon remission (Sharma et al., 2015). However, quality of life (QoL) does not always return to premorbid levels (Santos et al., 2012; Ragnarsson and Johannsson, 2013). To address this persistent impairment in QoL, several questionnaires have been developed to assist medical doctors and researchers working with patients with CS. To illustrate the use of person fit statistics in outlier detection, an example of a QoL questionnaire—the CushingQoL—for patients diagnosed with CS will be used.

The CushingQoL is the most commonly used disease-specific health-related QoL questionnaire designed for patients diagnosed with CS (Webb et al., 2008). Webb et al. (2008) developed the CushingQoL using standardized interviews with patients and endocrinologists. The single total score of the CushingQoL has demonstrated good construct validity (Santos et al., 2012; Badia et al., 2013; Roset et al., 2013), test–retest reliability (Nelson et al., 2013), and internal consistency (Santos et al., 2012). Recent research indicates the CushingQoL better represents patients QoL concerns when scored with two subscales: psychosocial issues and physical problems (Tiemensma et al., 2016). In addition to implementing this new scoring system, there are subsequent issues to explore linked to how well individual patients are represented by this scoring solution. In particular, it is important to highlight any patients that qualify as outliers, as the scoring system may not be representative of their QoL. Detection of outliers is an important component to consider when analyzing data in any context. Typically, outlier detection is implemented on the total score or subscale scores of a questionnaire. In this context, outliers represent patients whose subscale scores are more extreme than those of the “typical” patients (Grubbs, 1969). An important drawback of outlier detection is that it will not identify patients with substantively or clinically different item response patterns as being “atypical.” Using the person fit approach, patients with “atypical” response patterns are identified, even if their total scores are comparable to patients with more “typical” response patterns. This person fit approach to outlier detection allows the practitioner to identify patients with very different, or “atypical,” health concerns. Therefore, the aim of the current study is to illustrate how to derive person fit (Z*h*) statistics to detect outliers in survey research.

## Materials and methods

### Participants

Patients in the present study were recruited by Tiemensma et al. (2016), who examined different scoring options of the CushingQoL. A short message with a web link to the online survey was distributed to patients treated for CS through the Cushing's Support and Research Foundation's (CSRF) listserv and Facebook page. Patients were eligible to participate if they were over 18 years of age and in remission from CS. Patients were asked to complete the CushingQoL (English version) and a demographics survey. A total of 397 patients participated, with missing data for only three patients (<1%). The final analyses included the 394 patients who completed the entire CushingQoL survey (*n* = 30 males, *n* = 350 females, and *n* = 14 unknown)^1^. The protocol was approved by the University of California, Merced Institutional Review Board, and all patients provided digital informed consent before completing the survey.

### Protocol

The online survey started with a digital consent form, which described the nature of the study. When patients read the form and agreed to participate, a subsequent web page was loaded. On the second page, patients had to indicate if they were over 18 years of age. If a patient indicated they met this criterion, then they were shown the next page where they were asked about remission status. If the patient confirmed to be in remission, then the next web page was loaded that included a set of instructions for how to fill out the CushingQoL questionnaire. If they understood the instructions, then the patient would click to the next page, which included the 12 CushingQoL items as displayed in the original paper-and-pencil version published by Webb et al. (2008). Upon completion of the survey, the patient clicked to the next page where they received a demographics survey to complete.

### CushingQoL

The most commonly used instrument to assess QoL in patients with CS is the Cushing's QoL questionnaire (CushingQoL; (Webb et al., 2008)). The CushingQoL is a disease-specific health related QoL questionnaire comprised of 12-items. Items are measured on a 5-point Likert-type scale assessing how often (Always to Never) or how much (Very Much to Not at All) each item has been related to the patient's CS in the previous 4 weeks. The CushingQoL provides insight into physical problems and psychosocial issues (Webb et al., 2008; Tiemensma et al., 2016). Scores for each of the subscales of the CushingQoL are summed and transformed to range from 0 (worst possible QoL) to 100 (best possible QoL).

### Graded response model (GRM) and model fit

The graded response model (Samejima, 1969, 1997) is an IRT model that was developed to evaluate surveys measured by ordinal responses such as ordered Likert-type scales. For this study, the multidimensional generalization of the GRM was used to evaluate a two-subscale (simple-structure) scoring solution of the CushingQoL. The factor structure was determined from a previous investigation of the CushingQoL dimensionality and scoring options (Tiemensma et al., 2016). This model characterizes item functioning by using two different types of parameters for each item; namely, the item discrimination and item difficulty parameters. Discrimination parameters (one estimated for each item) evaluate how well an item differentiates between individuals scoring high and low on a latent ability (e.g., QoL). Item difficulty captures the location between two adjacent categories in the latent trait metric (e.g., QoL). The number of difficulty parameters estimated corresponds to the number of response categories minus one present for a given item.

There are many factors that contribute to the detection of a person fit outlier, including: the type of misfitting response pattern, test length, latent trait levels, and model fit (Meijer and Sijtsma, 2001). Each participant's response pattern contributes to the overall model fit (Reise and Widaman, 1999). The fit statistic used in this paper was the reduced *M2*. Model fit in IRT models is evaluated through contingency tables of response patterns. When sample sizes are small, then it is possible that some of the cells in the contingency table will have too few (including zero) responses to accurately estimate model fit. The reduced *M2* is a limited-information fit measure that outperforms full-information fit statistics (like the Pearson χ^2^) when there are cells in the contingency table with few or no cases (Cai and Hansen, 2013). The reduced *M2* indicates an adequately fitting model when the *p* > 0.05, and it is expressed as:

(1) $$\begin{array}{l} M_{2}^{\text{*}}=N\left(p-\hat{\pi }\right)\prime C\left(p-\hat{\pi }\right), \end{array}$$

where *N* is the sample size, *p* is a vector of observed response probabilities, $\hat{\pi }$ is a vector of model implied response probabilities, and *C* is a weight matrix of response patterns. The weight matrix is specified as n* ∑(*m*), and it is defined by a pattern of 0's and 1's indicating the relationship between the response pattern and the location of the item used for the computation of the first or second moment. The reduced *M2* statistic is asymptotically χ^2^ distributed with degrees of freedom equal to $\left(\left[m+\frac{m\left(m-1\right)}{2}\right]-\xi \right)$, where: *m* represents the total number of first moments, (*m*(*m* − 1))/2 is the total number of second moments, and ξ represents the total number of estimated model parameters. Once overall fit of the model is established, then person fit can be examined (Maydeu-Olivares and Joe, 2005).

### Person fit

Person fit is a broad set of statistical methods used to identify response patterns that are deemed unlikely to be observed based on the model. When a response pattern is deemed unlikely, then it is assumed that the responses to the survey items are guided by a response mechanism other than the construct specified (Meijer, 2002). For example, a person randomly selecting a response to items in order to get to the end of the questionnaire faster would produce person-misfit since the mechanism of selecting items is guided purely by guessing.

Person fit can have multiple uses including the detection of outliers (Meijer, 2002). There has been a growing interest to not only identify outliers, or so-called “misfitting” response patterns, but to also understand the reason for this response (Reise, 1990; Meijer, 2002, 2003; Meijer et al., 2008; Conijn et al., 2014). Several researchers have examined the reasons for such response patterns to appear across different contexts, including typical and maximum performance (Ferrando and Chico, 2003; Meijer et al., 2008; Conijn et al., 2014). In maximum performance settings (e.g., taking a math test), misfitting may occur due to a myriad of different reasons, including guessing, cheating, or taking a different approach to answering questions. In the context of health measures (i.e., typical performance), misfitting can occur due to fluctuating answers across domains (e.g., if a patient is experiencing severe issues in one aspect of QoL but not in others), distraction, low motivation, and exaggerating good/bad (Reise and Waller, 1993; Ferrando and Chico, 2003; Meijer et al., 2008; Ferrando et al., 2016). Research on the different uses of person fit has highlighted its importance and diagnostic value, and this statistical assessment is quite relevant to assessing QoL patterns in patients.

The *Zh* statistic is a standardized person fit value of *lz*, which is generalized to categorical data (Drasgow et al., 1985). The categorical *lz* statistic can be defined as:

(2) $$\begin{array}{l} lz=P\left(Y_{i}\text{|}\theta_{i}\right)=\sum_{i=1}^{n}\sum_{j=1}^{A+1}\delta_{j}\left(v_{i}\right)\log Pg\left(\theta \right), \end{array}$$

where *Y*_*i*_ represents the item responses, θ_*i*_ is contains the latent trait estimates, *n* represents the sample size, *A* is the number of *j* categories in item *i*. The random vector of item choices, denoted as δ_*j*_(*v*_*i*_), is used to ensure that only the probabilities of the chosen responses are summed. Thus, δ_*j*_(*v*_*i*_) is set equal to 1 when *j* = *k*, and it is set to 0 when *j* ≠ *k*. Finally, the standardized form of *lz* can be defined as *Zh* with the following transformation:

(3) $$\begin{array}{l} Zh=\left[lz\left(\theta \right)-E\left(lz\left(\theta \right)\right)/SD\left(lz\left(\theta \right)\right)\right], \end{array}$$

where *E*(*lz*(θ)) represents the mean *lz*-value for the sample, and *SD*(*lz*(θ)) represents the standard deviation. This transformation simply standardizes the value to have a mean of 0 and variance of 1 by dividing the difference between *lz* and mean of *lz* with the standard deviation of the observed *lz*-value.

**Figure 1:**
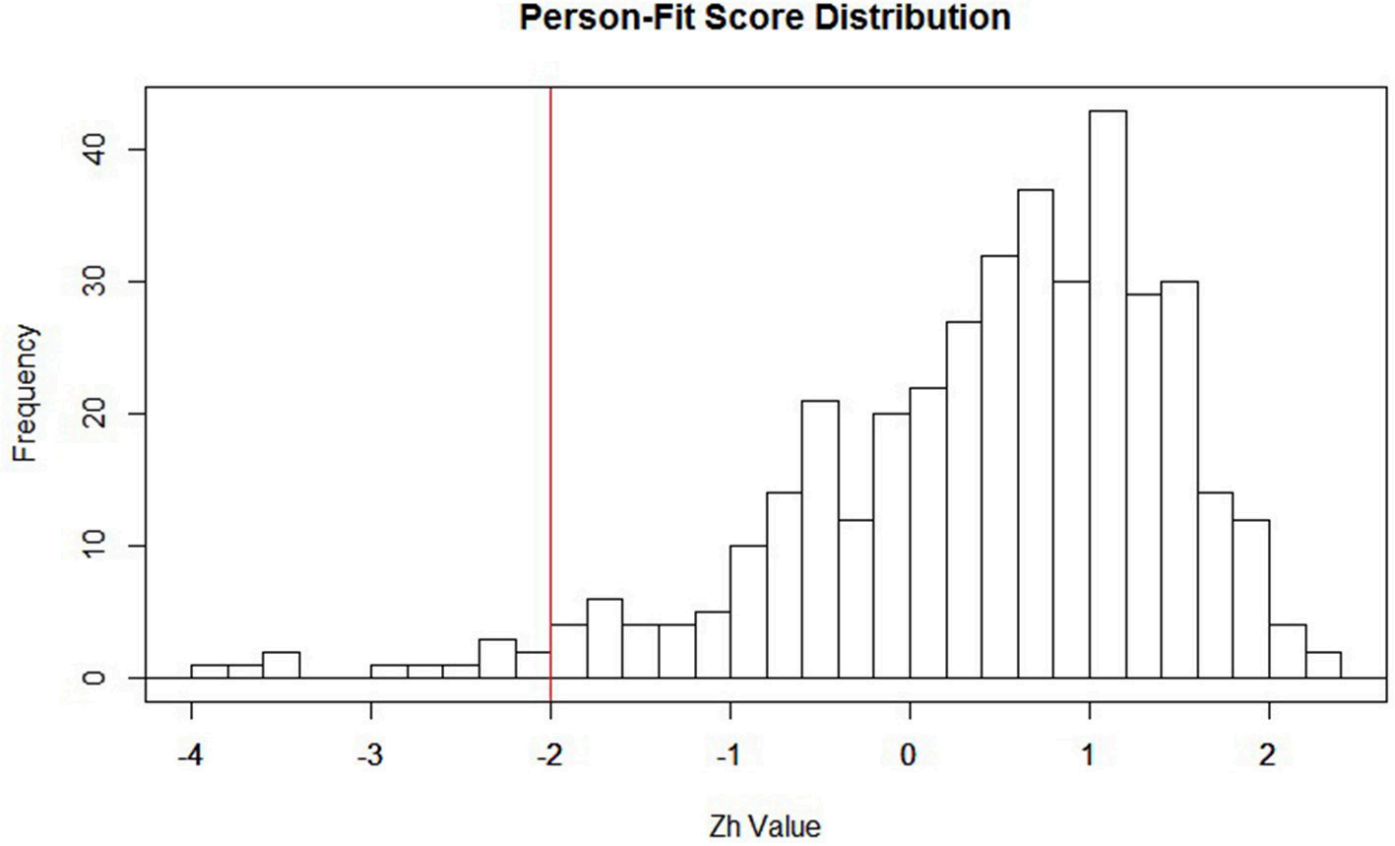
**Distribution of ***Zh*** values with −2 highlighted in red**. This is not a strict cutoff; it is suggested researchers create a cutoff that best highlights important features of the distribution. For example, if the interest is in assessing over-fit, then the cutoff would be placed at the higher end of the distribution (e.g., around 2).

Within the context of IRT, estimates are obtained for the individual item parameters, as well as the latent trait (i.e., QoL). When these parameter values are estimated within the model, the *Zh* distribution is then formed. Typically, the distributional form for *Zh* is non-normal, setting it apart from the more familiar normally distributed *z*-statistic (Drasgow et al., 1985). Due to the non-normality of the *Zh* distribution, a hard cutoff of −1.96 (akin to what is used with the conventional *z*-statistic) should not be used for making inferences. Rather, this cutoff of −1.96 can be viewed as a starting point to identifying misfitting responses. It may be that this value of −1.96 is altered once the substantive meaning of patients classified as outliers is thoroughly examined. After visually inspecting the distribution of *Zh*-values in the current study (see Figure 1), we chose to form a cutoff of ±2.0 to serve as the starting point in identifying aberrant responses for our participants. We also settled on the ±2.0 cut-off to mimic familiar conventions with the *z*-distribution. Participants with *Zh*-values above or below the cutoff do not necessarily reflect outliers, but rather participants with “atypical” response patterns that warrant further inspection. Researchers employing this method as an exploratory approach may also find it useful to start with a larger cut-score (say ± 3) and move from there as the aberrancy of responders falling in that extreme may be clearer.

The graded response model, overall model fit, and person fit were all estimated in the R programming environment (R Development Core Team, 2008) with the multidimensional IRT package (MIRT; Chalmers, 2012)^2^. We provide a description of how to implement this model, as well as annotated code.

## Results

### Patient characteristics

Two hundred and seventy-two patients (69%) received a Cushing's disease diagnosis, 97 patients (25%) were diagnosed with Cushing's syndrome, and six patients (1.5%) were diagnosed with medication-induced Cushing's syndrome. Twenty-five patients were not sure what their exact diagnosis was. Two-hundred and sixty-three patients (67%) underwent transsphenoidal surgery, 77 patients (20%) underwent unilateral adrenalectomy, and 65 patients (17%) bilateral adrenalectomy. Twenty-seven patients (7%) received post-operative radiotherapy. All patients reported being in remission, with a mean duration of remission of 6.7 years (*SD* = 7.4 years) and a mean follow-up duration of 8.7 years (*SD* = 8.3 years). One hundred and fifty-two patients (39%) reported some form of hypopituitarism and 169 patients (43%) reported being on Hydrocortisone substitution therapy with an additional 68 patients (17%) using Fludrocortisone.

### GRM model fit and parameter estimates (Table 1)

Because the focus of the current paper is on the ability to detect patients with irregular response patterns, GRM parameter estimates will only be briefly discussed. The two-subscale scoring solution of the CushingQoL reflected the data well, *M2*_(17)_ = 26.57, *p* = 0.065. See Table 1 for full results of the IRT parameter estimates. This table contains information on item discrimination (also called *slopes*), which is denoted in Table 1 as “a1” and “a2”; these represent discrimination levels for each of the two subscales, respectively. Item intercepts reflecting the different response categories for the items are denoted in Table 1 as “d1–d4”; converted to −(d/a), these estimates represent the difficulty estimate between response categories 1 and 2 (d1), between response categories 2 and 3 (d2), between response categories 3 and 4 (d3), and between the final response categories 4 and 5 (d4). Finally, Table 1 contains the factor loadings obtained from the analysis; these are denoted as “Load1” and “Load2” for the two subscales, respectively. Each item was able to discriminate between high and low QoL for each subscale of the CushingQoL (all slopes >1.03). Item difficulty parameters indicated that patients with more impaired QoL were more likely to select the lower response options for each item, whereas patients with higher QoL were more likely to select the higher response options. As previously mentioned, the response categories (d1–d4 in Table 2) can be converted to difficulty parameters (see Embretson and Reise, 2000) and interpreted as the point at which a person with certain ability or trait will endorse the higher of two competing item response categories. For example, the first intercept estimate (d1) for question 1 (“I have trouble sleeping”) is 1.034, converted to a difficulty parameter yields a value of −1.69; using the conversion of –(d/a) the computation would be (−1.748/1.034 = −1.69). A participant with a score of −1.69 on the psychosocial subscale would have a 50% chance of endorsing category 2 (“Often”) over category 1(“Always”) but >50% chance of endorsing category 3 (“Sometimes”). A participant with a score below −1.69 is more likely to select the lowest category (“Always”). For a review on how to fully interpret IRT parameter estimates, see Embretson and Reise (2000). We now turn our attention over to the person fit results to aid in determining whether outliers were present.

**Table 1:** **Item parameter estimates**.

	**a1**	**a2**	**d1**	**d2**	**d3**	**d4**	**Load1**	**Load2**
Cush1	–	1.034	1.748	0.146	−1.314	−3.380	–	0.719
Cush2	1.809	–	2.285	1.017	−0.396	−2.174	0.875	–
Cush3	–	3.819	5.261	2.564	−0.772	−3.875	–	0.967
Cush4	–	2.059	2.858	1.143	−0.806	−2.842	–	0.900
Cush5	1.403	–	2.981	1.173	−0.640	−3.407	0.814	–
Cush6	2.248	–	2.321	0.480	−1.537	−3.685	0.914	–
Cush7	2.067	–	1.239	−0.193	−1.702	−3.673	0.900	–
Cush8	2.634	–	3.052	0.417	−1.331	−3.739	0.935	–
Cush9	3.233	–	2.850	0.847	−1.374	−3.645	0.955	–
Cush10	3.268	–	2.358	0.676	−1.427	−3.931	0.956	–
Cush11	1.593	–	1.812	−0.359	−2.677	−4.598	0.847	–
Cush12	1.697	–	0.561	−0.995	−3.490	−5.433	0.862	–

*M2_(17)_ = 26.57, p = 0.065. RMSEA = 0.038 [0, 0.064], a1–a2 = item discrimination parameters (slopes) for the two respective factors, d1–d4 = intercept parameters. The intercept estimate between response categories 1 and 2 (d1), between response categories 2 and 3 (d2), between response categories 3 and 4 (d3), and between the final response categories 4 and 5 (d4). Load1−Load2 = factor loadings for the two subscales, respectively. Based on the results, all items were able to discriminate between high and low QoL for each subscale of the CushingQoL (i.e., all slopes > 1.03). Item intercept parameters indicated that patients with more impaired QoL were more likely to select the lower response options for each item, whereas patients with higher QoL were more likely to select the higher response options*.

**Table 2:** **Person fit values and their corresponding response patterns for most misfitting, well fitting, and overfitting persons**.

**ID**	***Zh* score**	**Q1**	**Q2**	**Q3**	**Q4**	**Q5**	**Q6**	**Q7**	**Q8**	**Q9**	**Q10**	**Q11**	**Q12**
**MISFITTING**													
**392**	−3.92791	**1**	2	**4**	**4**	4	5	4	3	3	2	5	5
**345**	−3.79973	**2**	5	**5**	**3**	2	2	1	1	5	5	3	1
**379**	−3.52911	**4**	3	**3**	**4**	4	5	3	1	5	3	5	4
**81**	−3.47521	**2**	5	**4**	**2**	1	1	2	1	5	2	1	1
**113**	−2.94507	**1**	1	**2**	**1**	1	5	4	4	2	2	1	2
**391**	−2.79318	**2**	1	**3**	**3**	3	4	4	5	1	2	4	1
**1**	−2.54869	**2**	1	**5**	**4**	4	3	5	2	2	1	2	4
**185**	−2.39281	**1**	2	**4**	**3**	1	1	3	5	1	1	1	1
**229**	−2.24675	**3**	3	**1**	**4**	3	3	4	3	3	1	3	4
**221**	−2.21036	**3**	3	**2**	**2**	4	3	2	4	3	1	5	4
**WELL FITTING**													
**48**	−0.03243	**4**	4	**5**	**4**	5	4	4	5	3	5	4	3
**130**	−0.02635	**2**	1	**3**	**3**	3	4	3	2	1	1	1	1
**302**	−0.00813	**4**	4	**2**	**1**	3	4	2	3	2	4	2	1
**43**	−0.0019	**4**	1	**4**	**2**	3	1	1	1	1	1	3	1
**256**	0.007965	**5**	5	**5**	**5**	4	2	5	5	5	5	3	3
**136**	0.02727	**5**	5	**4**	**3**	4	5	5	5	5	5	5	5
**111**	0.02917	**1**	5	**2**	**1**	3	1	1	3	2	3	1	1
**202**	0.033304	**4**	3	**5**	**5**	4	4	4	4	3	2	1	1
**297**	0.036985	**4**	2	**1**	**1**	2	1	1	1	1	1	1	1
**55**	0.041895	**4**	4	**5**	**5**	5	3	5	5	3	4	3	3
**OVERFITTING**													
**157**	1.944592	**3**	4	**3**	**3**	4	3	3	2	3	3	3	3
**389**	1.984945	**2**	3	**2**	**2**	3	2	2	2	2	2	2	2
**67**	1.98928	**2**	3	**3**	**3**	3	3	4	3	3	3	2	2
**128**	1.99864	**3**	4	**4**	**3**	4	3	2	4	4	4	3	3
**162**	2.070151	**3**	4	**3**	**3**	3	3	3	4	4	3	3	3
**331**	2.098399	**4**	4	**5**	**5**	3	3	2	3	3	3	3	2
**235**	2.155638	**3**	4	**3**	**3**	4	4	3	3	4	4	3	3
**362**	2.196376	**2**	3	**3**	**3**	3	3	2	2	2	2	2	1
**380**	2.202284	**2**	5	**4**	**3**	4	4	4	4	4	4	3	3
**320**	2.221422	**3**	4	**5**	**4**	4	4	3	4	4	4	3	3

*Bold font represents items relating to the second factor. Zh-values can be interpreted using the z-scale distribution. For example, a respondent with a Zh-value of −1 shows a response pattern that deviates from the average response by one standard deviation. In addition, a respondent with −2 indicates his or her response deviates by two standard deviations from the average response pattern*.

### Person fit results (Table 2)

When investigating outliers using traditional methods (i.e., box-plots), zero outliers were detected. While the traditional outlier detection methods revealed no outliers, person fit statistics detected 18 patients with “atypical” response patterns. Specifically, there were 12 patients with misfitting responses (*M* = −2.84, *SD* = 0.69), and six patients with overfitting response patterns (*M* = 2.15, *SD* = 0.61). Table 2 lists the response pattern for each individual item on the CushingQoL for the 10 patients with the highest misfitting scores (i.e., *Zh* score lower than −2). For comparison, 10 well-fitting patients were included (i.e., those with *Zh*-values close to zero) and 10 overfitting patients (i.e., highest positive *Zh*-values). Since the *Zh*-values are standardized, they can be interpreted as *z*-score values.

A closer inspection of patients with misfitting response patterns typically selected the extreme response options (i.e., they often endorsed “always” or “never”). This could indicate that these patients have more severe problems with certain aspects of QoL, and no or very little problems with others. It is important to note that these “atypical” patients showed similar subscale scores compared to the whole sample, and these “atypical” patterns would normally go unnoticed using conventional outlier detection methods. Patients with overfitting response patterns typically selected the middle response options (i.e., they often endorsed “sometimes” or “somewhat”). Patients with extreme person fit statistics did not differ on any measured covariate and therefore would not have been detected using traditional outlier detection methods, underscoring the importance of this method.

## Discussion

The aim of the current paper was to highlight how to detect “atypical” response patterns of participants using the accessible and free R statistical software programming environment. Person fit statistics were generated from the default settings of the R package, mirt. This package uses an IRT likelihood-based approach to detect participants with “atypical” response patterns. In contrast to conventional outlier detection techniques, person fit statistics provide insight into the exact pattern of responses across items. It is important to properly identify participants with different response patterns. The exact pattern of responses across items can provide researchers with richer and more precise results related to various psychological constructs for each individual.

In the present study, the items on the two subscales of the CushingQoL were able to adequately discriminate between patients with better vs. more impaired QoL. In our assessment of outliers, a conventional method (i.e., box-plots) indicated no outliers in the dataset. However, person fit statistics unveiled several patients with “atypical” response patterns. This finding illustrates the value and importance of the person fit approach.

Implementing person fit statistics can be helpful for researchers to detect participants who have “atypical” response patterns, but who appear “average” when examining their total score or subscale scores on questionnaires. It is important to note that participants falling out of the bounds of the *Zh* cutoffs are not necessarily “problematic.” Rather, these cutoff points can be used as a starting point for identifying *potentially* “problematic” subjects within the current sample, or participants who may warrant further investigation. The arbitrary cutoff-values of ±2.0 were used to provide an intuitive starting point, and they were not meant to act as a hard cutoff for flagging “problematic” cases. It is the researcher's responsibility to determine whether these cases are acting as outliers or not. We recommend investigating a histogram of the *Zh* statistics to determine an appropriate cut-off point to use as a diagnostic for person fit outlier detection. Because the distribution of the *Zh* statistic is not well-understood, and may not always approximate a normal distribution, the cutoff-values should be determined at the discretion of the researcher based on the goals underlying the identification of cases.

Although the person fit method is uncommon in the applied psychology literature, it is relatively easy to implement and interpret. Furthermore, person fit statistics can be estimated for any type of questionnaire. This paper expands upon the goal of Ferrando et al. (2016) to incorporate the use of person-level outlier detection in applied research. We provide an alternative method from an IRT likelihood-based approach in an accessible and free statistical software program. We included annotated code in Table 3 to aid researchers in using this method. Data used in this example will also be provided as an online supplement.

**Table 3:** **Statistical Code for outlier detection using the Person Fit method**.

**Code**	**Annotation**
https://cran.r-project.org/	First, you have to download and install the free R program. Just copy and paste the URL in the left column to your web browser. The following code (in the left column) can all be copy and pasted into the R script after it is downloaded.
install.packages(“mirt”) library(mirt)	Once you have downloaded R, you have to install and load the package to estimate item response theory models. The install.packages(“mirt”) command tells R that you want to install the multidimensional item response theory (mirt) package. After doing this, R will ask you to select a CRAN mirror (i.e., location to download the program from). Just select the location closes to where you are implementing the analysis. After the package has installed, you can load it to R using library(mirt).
data <- read.csv(“C:/Users/name/desktop/Dataset.csv,” header = T)	Next, you will have to load your data into R. Data files can be uploaded from a variety of files including .txt, .csv., SPSS data files, SAS data files, or Stata data files (see http://www.statmethods.net/input/importingdata.html). To load the data, you want to put the full path name to the file saved on your computer within quotation marks. If the variable names are included at the top of your data set, make sure to set header = T so R knows the first row of the data file is variable names. We saved our data to an object called “data” for ease of coding later on (object is to the left of the arrow, where the arrow means the object the arrow is pointing toward gets what is on the other side of the arrow.
cfa2 <- mirt.model(“P = 2, 5–12 S = 1, 3, 4 COV = P^*^S”)	This line of code is how you specify which items belong to which subscale. If your questionnaire is just a single total score, skip this part. Here, we saved our subscales to the object we called “cfa2.” We specified the Physical subscale (P) as containing items 2, and 5–12, and the PsychoSocial subscale(S) as containing items 1, 3, and 4. We also indicated that both subscales are correlated with the COV = P^*^S command.
mod1 <- mirt(data, cfa2, itemtype = “graded”)	This line of code is how you estimate the item response theory model. We have saved the model to an object called mod1. Within the mirt command, the first thing you do is specify your data set. Here, we put “data” because that is what we named our dataset earlier. Next, we specify any subscales (cfa2). If your questionnaire has no subscales, you would put the number 1 instead, indicating a single score. itemtype = “graded” indicates that the items are ordered categorical (i.e., Likert-type scales). If the items on your questionnaire only contain two responses, you would specify itemtype = “2PL.”
pfit <- personfit(mod1)	The person fit function allows you to generate person fit scores for each subject. Here, we saved this information in an object we called pfit.
PFdata <- cbind(pfit, data)	Next, we wanted to add the person fit scores for each subject to the data set. To do this, we used the cbind function in R to add the column of person fit scores to the original data set. We then saved this combined dataset to the object PFdata.
sorted.dat <- PFdata[order(PFdata[,1])	After combining the person fit scores to the data files, you will want to inspect the data. To make this easier, you can sort the data by the person fit scores (*Zh* values) using the code to the left. Here, we specify the PFdata will be ordered from lowest to highest by the first column of the data set (as seen in the [,1]). If the scores are in a different column in your dataset, then you can change the 1 to the corresponding column number.
head(sorted.dat,20) tail(sorted.dat,20)	You can choose to look at the most misfitting response and the most “overfitting” responses using the head or tail commands. Here, we specified the first 20 responses (head) and the last 20 responses (tail).
hist(sorted.dat[,1])	You can look at a histogram of your person fit scores (*Zh*). The distribution should be relatively normal. If there is a heavy negative skew, you have evidence of “misfitting” responses that merit further investigation.

In summary, the person fit method has proved to be a valuable tool in detecting clinically important but “atypical” patients with Cushing's syndrome. Out of 394 patients, 18 patients were classified as “atypical” using this process. These patients would have gone unnoticed with conventional outlier methods. However, the person fit method allows researchers and medical professionals to identify and better treat patients. This method is generalizable to any type of research question where survey data is collected.

## Author contributions

JF: Co-designed the study, co-collected the data, co-developed the idea for this study, ran all statistical analyses, co-created all tables and figures, substantial writing and editing; RC: Co-developed the idea for this study, ran all statistical analyses, co-created all tables and figures, substantial writing and editing. JT: Co-designed the study, co-collected the study, co-advisor on project, substantial writing and editing. SD: Co-advisor on project, ran all statistical analyses, substantial writing and editing.

### Conflict of interest statement

The authors declare that the research was conducted in the absence of any commercial or financial relationships that could be construed as a potential conflict of interest.

## Footnotes

^1^We conducted a small simulation study to gauge how accurately the model could be estimated under a sample size of 394 with similar parameter values to our empirical data. Specifically, we generated data with known parameter estimates based on parameters from our QoL sample. We generated 1,000 datasets under these conditions (i.e., the simulation investigation contained 1,000 replications). Data generation and estimation were conducted in R; estimation was implemented in the mirt package using the EM algorithm (Chalmers, 2012). The results of this simulation indicated that a sample size of 394 accurately and consistently estimated the item parameters and latent trait covariance. This simulation specified conditions comparable to the QoL data in the present study. The results for this simulation component can be found at: https://www.dropbox.com/s/yb8kpm9qeyt6rxy/MiniSim%20Results.docx?dl=0.

^2^The mirt package provides multiple estimation options, but the default which we elected to use was the EM algorithm as it proves to be a consistent estimator for the 2PL and GRM models.
